# Matched case-control study of the influence of inland waters surrounding poultry farms on avian influenza outbreaks in Japan

**DOI:** 10.1038/s41598-018-21695-1

**Published:** 2018-02-19

**Authors:** Yumiko Shimizu, Yoko Hayama, Takehisa Yamamoto, Kiyokazu Murai, Toshiyuki Tsutsui

**Affiliations:** Viral Disease and Epidemiology Research Division, National Institute of Animal Health, National Agriculture and Food Research Organization, Tsukuba, Japan

## Abstract

To successfully control highly pathogenic avian influenza (HPAI), understanding the risk factors related to the incursion of the virus into poultry farms is essential. In this study, we focused on the presence of inland waters surrounding poultry farms as a potential risk factor of incursion of the virus. To evaluate the influence of inland waters surrounding poultry farms on HPAI outbreaks in Japan, a simple matched case-control study was conducted. The results of the conditional regression analyses indicated that the number of farms with neighbouring inland waters was significantly high among the affected farms during the 2016–2017 outbreak period. These results provide good grounds for strengthening biosecurity management at farms located near inland waters.

## Introduction

Highly pathogenic avian influenza (HPAI) is one of the most important diseases occurring in poultry industries owing to its high mortality and potential threat to public health. The disease has become a global concern since the emergence of the HPAI virus subtype H5N1 in Asia in 2003, which swiftly spread worldwide^1,2^. By August 2017, the disease has affected over 70 countries^3^ and several viral subtypes, including H5N1, H5N6, H5N8, and H7N9, circulate in various countries and regions^4^.

In Japan, an H5N1 HPAI outbreak first occurred in poultry farms in 2004, and HPAI has occurred nearly every year since, mainly during the winter season. In recent years, 24 (H5N1), 1 (H5N8), 5 (H5N8), and 12 (H5N6) cases occurred from November 2010 to March 2011, in April 2014, from December 2014 to April 2015, and from November 2016 to March 2017, respectively^2,5^.

To control the disease, it is necessary to know the risk factors related to the incursion of the virus into farms, and apply appropriate preventive measures. Previous reports have suggested several invasion routes, such as movement of live poultry between farms, and visitors and vehicles coming into farms^6–8^. There are also studies indicating the role of waterfowl as reservoirs of avian influenza viruses^9^ and the association between the viral migration and waterfowl migration routes in Asian region^10,11^. The risk factors related to HPAI outbreaks have been studied using statistical methods including case-control study^12–14^ and spatial modeling^15–18^. In those studies, inland waters including lakes and wetlands usable by waterfowl were considered as one of the important risk factors of disease spread in the surrounding area. If waterfowl carry viruses, inland waters could become a site of infection of the HPAI virus to other vectors (e.g., resident birds or rodents) or fomites carrying the virus into nearby farms^19–21^.

In our study, we focused on the evaluation of the influence of inland waters surrounding poultry farms on HPAI outbreaks in Japan. The reason why we focused only on the inland waters was that the HPAI outbreaks in Japan in 2016–2017 seemed to have occurred mainly at farms near inland waters, according to the on-site epidemiological investigations^22^. To evaluate the influence of inland waters, we conducted a matched case-control study of the inland waters surrounding farms, and compared the results of two previous outbreak periods: 2010–2011 and 2016–2017.

## Results

In total, 23 case farms and 89 control farms were subject to analysis for the 2010–2011 outbreak period, and 10 cases and 34 controls were analysed for the 2016–2017 outbreak period.

The distance from the farms to their nearest inland waters ranged from 8–402 m for the case farms and 2–1,469 m for the control farms affected during the 2010–2011 period, and 10–307 m in cases and 1–1,017 m in controls affected from 2016–2017. The measured distances are listed in Supplementary Tables S1 and 2.

When using a 50-m threshold to judge the presence of inland waters around the farms, 8 of 23 case farms in the 2010–2011 period had neighbouring inland waters, whereas 27 of 89 control farms had neighbouring waters. For the 2016–2017 outbreak period, neighbouring inland waters located within 50 m of the farms were observed for 9 of 10 case farms and 7 of 34 controls.

**Figure 1:**
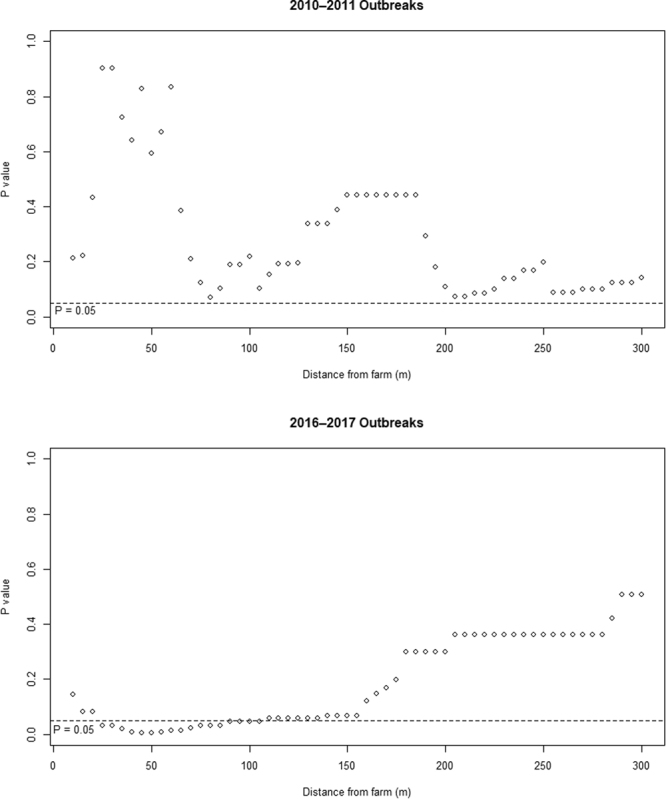
P values of the Wald tests obtained as results of the conditional logistic regression analysis. The presence of neighbouring inland waters around the farms was judged by each distance from the farm.

The results of the conditional logistic regression analysis by distance from the farms are shown as P values of Wald tests (Fig. 1). When P values < 0.05 were considered statistically significant, none of the results from the 2010–2011 outbreak period showed significant differences in the distance from inland waters between case and control farms. Conversely, for the 2016–2017 outbreak period, there were significant differences between the case and control farms when neighbouring inland waters were present within 25–105 m of the farms. Additionally, the results from 110–155 m were marginal (P = 0.06–0.07).

## Discussion

For the 2016–2017 outbreak period, the presence of neighbouring inland waters was significantly associated with the occurrence of HPAI on poultry farms in Japan. A significant difference was observed for distances of 25–105 m from inland waters consistently; a significance was not observed for distances <25 m and >105 m. When distance thresholds of >105 m were applied, inland waters were identified around many farms. Meanwhile, when the distance was <25 m, few farms had neighbouring inland waters. Inland waters surrounding poultry farms have been considered one of the risk factors of HPAI outbreaks since they provide suitable habitats for wild animals, including waterfowl^12–14,17^. Waterfowl migrating to inland waters may directly contaminate nearby farms, or contaminate the environments near inland waters, and small animals, such as resident birds and rodents, or humans may carry the viruses once they have been in contact with those contaminated environments and farms^19–21^.

In contrast, during the 2010–2011 outbreaks, the presence of neighbouring inland waters around farms was not significantly related to the occurrence of HPAI. This discordance may be explained by the assumed higher biosecurity at poultry farms in 2016–2017 season than in 2010–2011. After the outbreaks of HPAI in the 2010–2011 season, the legal standard for biosecurity measures at poultry farms was strengthened to require farmers to manage the entrance of vehicles and humans into farms, to disinfect those entering farms, and to cover poultry houses with netting for protection from wild birds. In the report from Japan’s Ministry of Agriculture, Forestry and Fisheries (MAFF), the proportion of chicken farms fulfilling all requirements of the legal standard for biosecurity measures at farms improved from 59.2% in 2011 to 76.8% in 2016^23^. Additionally, despite the number of HPAI-infected wild birds being much greater in 2016–2017 (218 cases) than in 2010–2011 (62 cases) thereby indicating a higher level of the HPAI virus in the environment in 2016–2017^22^, the number of affected farms in the 2016–2017 outbreak (12 farms) was less than that of the 2010–2011 outbreak (24 farms). These may suggest that the biosecurity at farms were improved in the 2016–2017 period, and therefore, the outbreaks were limited to farms that were exposed to additional risks, such as those located nearby inland waters.

This study had some limitations. First, we considered rivers, irrigation canals, ponds, and lakes to all be inland waters. The detailed conditions of these inland waters, such as water volume, size of the water’s surface, speed of the water stream, freezing conditions, etc., were not considered. Second, on-site observation of waterfowl and other wild animals around these inland waters was not conducted. However, the habitat choice of wild animals is influenced by several factors, including the type of inland water and its surrounding environment. Therefore, the risk of the presence of inland waters as defined by this study may include various pathways of exposure, both into and from these water bodies, with different likelihoods. Given the limited number of case farms and information sources available for the most current outbreaks, conducting a detailed analysis of the risk of inland waters was not possible in this study. Further investigation regarding the exposure mechanisms of poultry farms may provide deep insight as to the risks associated with nearby inland waters.

In conclusion, this study demonstrated that inland waters surrounding poultry farms are a potential risk factor for the occurrence of HPAI outbreaks. These findings strongly suggest that poultry farms with nearby inland waters (as opposed to farms without) should take further biosecurity measures to prevent the incursion of HPAI, and if possible, to restrict access of waterfowl to nearby inland waters (e.g., by covering ponds with bird-control netting).

## Methods

### Study design and data collection

A matched case-control analysis was conducted for farms affected by HPAI from 2010–2011 and 2016–2017, periods when a relatively large number of farms were affected. For the analysis, each affected farm was considered a case farm. Control farms corresponding to each case farm were selected from farms raising ≥ 5,000 poultry, and the types of control farms (i.e., layers or broilers) were matched with the case farms. Breeder farms were treated as layer farms because the management practices are similar in Japan. We planned to select four control farms for each case farm to ensure the power of the test^24^. The control farms were selected within a radius of 20 km from each case farm to obtain a sufficient number of control farms at similar geographical situation including distribution of habitats of wild birds. When the number of available control farms was less than four, all were used as controls. Two affected farms in the 2016–2017 outbreak period and one in the 2010–2011 period were excluded as case farms because no control farms were available due to low farm densities in these areas.

For every case and control farm, inland waters around the farm were identified by visual observation of aerial photographs using Google Earth Pro. Rivers (including irrigation canals), ponds, and lakes were identified as inland waters. When the aerial images were not clear, map images from Google Maps were supplementally used to identify inland waters. The direct distance between the nearest inland water and any of the poultry houses on each farm was measured using the ruler function in Google Earth Pro.

Although we used aerial photographs that were taken at dates as close as possible to each outbreak season, the dates varied from May 2006 to April 2015 for the 2010–2011 outbreak data, and from June 2014 to February 2017 for the 2016–2017 outbreak data. However, on-site investigations were conducted by veterinary authorities for all affected farms, the results of the investigations of the presence of inland waters were compared, and consistency was confirmed with the results observed from the aerial photographs obtained from Google Earth Pro.

### Statistical analysis

To assess whether the influence of inland waters as a risk factor of HPAI outbreaks is affected by the distance between farms and inland waters, we converted the distance into a dichotomous variable by setting thresholds to judge the presence of inland waters surrounding farms. The threshold values were changed from 10 m to 300 m, by 5-m increments. The presence of inland waters surrounding farms was adopted as the explanatory variable, and the occurrence of HPAI at each farm was defined as the objective variable. Case numbers attached to each of the case/control farms were treated for grouping to apply a conditional regression analysis. All statistical analyses were conducted using R (R Core Team [2013]. R: A language and environment for statistical computing. R Foundation for Statistical Computing, Vienna, Austria. ISBN 3–900051–07–0, http://www.R-project.org/). For the conditional logistic regression analysis, the clogit function from the R ‘survival’ package^25,26^ was used.

### Data availability

All data generated or analysed during this study are included in this published article (and its Supplementary Information files).

## Electronic supplementary material

Supplementary Tables
